# Tristetraprolin mediates immune evasion of mycobacterial infection in macrophages

**DOI:** 10.1096/fba.2024-00022

**Published:** 2024-06-29

**Authors:** Jiawei Wei, Huan Ning, Octavio Ramos‐Espinosa, Christopher S. Eickhoff, Rong Hou, Qinghong Wang, Mingui Fu, Ethan Y. Liu, Daping Fan, Daniel F. Hoft, Jianguo Liu

**Affiliations:** ^1^ Division of Infectious Diseases, Allergy and Immunology, Department of Internal Medicine Saint Louis University School of Medicine, Saint Louis University St. Louis Missouri USA; ^2^ Shock/Trauma Research Center, Department of Basic Medical Science, School of Medicine University of Missouri‐Kansas City Kansas City Missouri USA; ^3^ Department of Cell Biology and Anatomy University of South Carolina School of Medicine Columbia South Carolina USA

**Keywords:** cytokine, immune evasion, Macrophages, RNA‐binding protein, tristetraprolin, tuberculosis

## Abstract

Immune evasion of *Mycobacterium tuberculosis* (Mtb) facilitates intracellular bacterial growth. The mechanisms of immune evasion, however, are still not fully understood. In this study, we reveal that tristetraprolin (TTP), one of the best characterized RNA‐binding proteins controlling the stability of targeted mRNAs, mediates innate immune evasion of mycobacteria. We found that TTP knockout mice displayed reduced bacterial burden in the early stage after Mtb aerosol challenge. Macrophages deficient in TTP also showed an inhibition in intracellular mycobacterial growth. Live mycobacteria induced TTP protein expression in macrophages, which was blocked by the mTOR inhibitor rapamycin. Rapamycin and AZD8055 specifically blocked 4EBP1 phosphorylation in infected macrophages and suppressed intracellular BCG growth. Rapamycin promoted TTP protein degradation through the ubiquitination pathway, whereas the proteasome inhibitor MG‐132 blocked rapamycin function and thus stabilized TTP protein. TTP induction suppressed the expression of iNOS/TNF‐α/IL‐12/IL‐23, and weakened protective immune responses in macrophages, whereas rapamycin enhanced the bactericidal effects through TTP inhibition. Moreover, blocking TTP binding increased the expression of TNF‐α and iNOS and suppressed intracellular mycobacterial growth. Overall, our study reveals a novel role for RNA‐binding protein TTP in Mtb immune evasion mechanisms and provides a potential target for host‐directed therapy against tuberculosis (TB).

## Graphical abstract

Immune evasion of *Mycobacterium tuberculosis* (Mtb) facilitates intracellular bacterial growth. In this study, we found that tristetraprolin (TTP) knockout mice and macrophages deficient in TTP displayed reduced bacterial burden after mycobacterial infection. Live mycobacteria induced TTP protein expression through mTORC1, specifically 4EBP1 phosphorylation in infected macrophages. Mtb‐activated mTOR signaling stabilized TTP protein through inhibition of the ubiquitination pathway. TTP induction suppressed the expression of iNOS/TNF‐α/IL‐12/IL‐23, and weakened protective immune responses in macrophages, leading to increased intracellular mycobacterial growth.

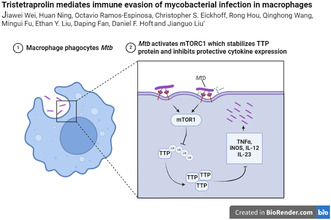

## 1 INTRODUCTION

Tuberculosis (TB) remains a leading global health problem with about 9 million new cases and nearly 1.5 million TB‐related deaths worldwide each year.^1^, ^2^, ^3^, ^4^ The lack of highly effective preventive vaccine, the emergence of drug‐resistant strains, and an epidemic in HIV patients make TB a worsening threat. Approximately 25% of the world's population is currently infected with *Mycobacterium tuberculosis* (Mtb) varied from asymptomatic or latent infection to activate disease.^5^ The diverse clinical outcomes of Mtb infection are mainly determined by different host immune responses.^6^, ^7^, ^8^ Mtb infection persists in macrophages when the host immune responses are suppressed or evaded.^9^, ^10^, ^11^, ^12^ The mechanisms of TB immune evasion, however, remain elusive. Macrophages play a crucial role in mycobacterial pathogenesis as both effector cells of the immune responses against Mtb infection and the host cells of Mtb. Different from extracellular bacteria, Mtb develops resistance to host innate immunity and manipulates host macrophages to establish an ideal replicative niche. A failure of macrophages to eliminate intracellular Mtb results in latent infection or granulomatous lesions.^13^ The innate immune responses and macrophages are mainly regulated by cytokines. Gene profiling studies have provided evidence for the importance of proinflammatory cytokines, such as TNF‐α, IFN‐γ, IL‐12, and iNOS, in host defense against Mtb infection.^14^ Alteration of these cytokine profiles and proinflammatory mediators during Mtb infection results in different outcomes, such as advancing or controlling infection.^15^ The mechanisms that regulate expression of these cytokines during mycobacterial infection, however, are still not fully understood.

Posttranscriptional control of gene expression is one of the major mechanisms in cytokine regulation, which allows for rapid changes in cytokine mRNA levels. Tristetraprolin (TTP), one of the tandem CCCH zinc finger (TZF) protein members, is involved in posttranscriptional regulation of inflammatory responses.^16^ The binding of TTP TZF domain to the adenosine and uridine‐rich elements (ARE) within the 3' untranslated region (3'UTR) of target mRNAs results in a rapid decay of mRNAs. Most of the cytokines important for TB pathogenesis are regulated by TTP, including TNF‐α,^17^ IFN‐γ,^18^ IL‐12,^19^ and iNOS.^20^ TTP acts like a resolution protein promoting mRNA decay of these inflammatory cytokines.^21^ Though TTP inhibits the cytokine expression, it is currently unknown whether Mtb could hijack TTP machinery to evade protective immune responses in infected macrophages.

The mammalian target of rapamycin complex (mTOR‐C) signaling pathway is a central regulator of cell metabolism, growth, proliferation, and survival.^22^ mTOR‐C consists of rapamycin‐sensitive mTOR complex 1 (mTOR‐c1) and rapamycin‐insensitive mTOR complex 2 (mTOR‐c2). The mTOR signaling pathway, a well‐known regulator of diverse immune cells, was found to extensively affect innate immune responses by enhancing the production of a series of proinflammatory cytokines, including TNF‐α, IL‐6, IL‐12, and IL‐23.^23^, ^24^, ^25^, ^26^ Accumulating evidence showed that some pathogens could alter immune responses or cell metabolisms through activating mTOR signaling pathway to facilitate their survival and blocking mTOR signaling exerted strong protective effects against infections induced by bacteria, virus, and parasite.^23^, ^24^, ^27^, ^28^, ^29^, ^30^, ^31^, ^32^, ^33^, ^34^ Though these proinflammatory cytokines regulated by mTOR signaling are also directly or indirectly regulated by TTP, the connection between TTP and the mTOR signaling pathway remains elusive.^35^, ^36^

In this study, we have shown that deletion of TTP in mice results in reduction of bacterial burden in the airway in the early stage of Mtb infection. Mycobacterial infection induces TTP protein expression in macrophages through the mTOR pathway via suppressing TTP protein ubiquitination. TTP induction by Mtb promotes decay of the protective proinflammatory cytokines and therefore weakens bactericidal effects of the infected macrophages. In line with these findings, blocking the mTOR pathway with rapamycin enhances bactericidal effects of the infected macrophages through suppressing TTP production. Our study reveals a novel role for RNA‐binding protein (RBP) TTP in TB immune evasion mechanisms and provides a potential novel target for host‐directed therapy against Mtb infection.

## 2 METHODS

### 2.1 Mice

The Zfp36^+/−^ mouse breeders were kindly provided by Dr. Perry J. Blackshear^37^ (National Institute of Environmental Health Sciences). All mice were on the C57BL/6 background and housed in cages with filter tops in a laminar flow hood, fed food, and water ad libitum at Saint Louis University (SLU) Animal Facilities in accordance with the principles of Animal Care (NIH publication number 85‐23, revised 1985). Animal experiments were approved by the Institutional Animal Care and Use Committee at SLU and were performed according to federal and institutional guidelines.

### 2.2 Mtb aerosol challenge

TTP^−/−^ mice and WT littermates aged 5–6 weeks were infected with Mtb via aerosol in BSL3 facility (~100 CFU of Mtb Erdman strain per mouse) using the Glas‐Col Inhalation Exposure System. Ten days post‐infection (dpi), lungs and spleens were taken and homogenized in 1 mL of PBS. Serial dilutions of lung and spleen homogenates were inoculated onto Middlebrook 7H10 agar plates and CFUs determined 3 weeks later. Each dot represents one mouse.

### 2.3 Cells and reagents

The murine macrophage cell line RAW264.7 (RAW cells) was obtained from American Type Culture Collection (ATCC) and maintained in RPMI 1640 supplemented with 2 mM glutamine, 100 units/mL of penicillin and streptomycin and 10% FBS (Sigma, St. Louis, MO, endotoxin NMT 10.0 EU/mL). RAW264.7 cells were authenticated by STR DNA profiling and detected for mycoplasma contamination by PCR DNA analysis. Mouse bone marrow cells were obtained from femurs as previously described.^38^ Bone marrow‐derived macrophages (BMDMs) were cultured in RPMI 1640 medium supplemented with 10% heat‐inactivated FBS (Sigma‐Aldrich, St. Louis, MO), 25 mM HEPES (Sigma‐Aldrich, St. Louis, MO), 2 mM glutamine (Sigma‐Aldrich, St. Louis, MO), 1 mM sodium pyruvate (Sigma‐Aldrich, St. Louis, MO), 100 U/mL penicillin–100 μg/mL streptomycin (Sigma‐Aldrich, St. Louis, MO) as well as 30% (v/v) L929 cell conditioned medium. After 7 days of culture, the fully differentiated and matured BMDMs were used for experiments.

### 2.4 Colony‐forming unit assay

BMDMs or RAW 264.7 cells were plated in 12‐well plates with density of 2 × 10^5^ cells per well. Cells were infected with BCG at MOI 3 for 1 h and then washed three times with RPMI‐1640 medium to remove extracellular BCG. Infected cells were incubated in full medium for an additional 48 h (BMDM) or 2 h (RAW 264.7). Cells were lysed with 1 mL of water. Quantitative culturing was performed using 10‐fold serial dilutions. Aliquots of 50 μL of each dilution were inoculated on Middlebrook 7H10 agar plates with 10% (v/v) OADC. Plates were incubated at 37°C for 3 weeks, followed by CFU counting.

### 2.5 BCG growth inhibition assay

BMDM or RAW 264.7 cells were plated in 96‐well plate with density of 1 × 10^5^ cells per well. Cells were infected with BCG at MOI 1–3 for 1 h and then washed three times with RPMI‐1640 medium to remove excess BCG. After 48 h of incubation, cells were lysed with 100 μL of 0.2% saponin and BCG was released from cells. One hundred microliters of 1:100 diluted 5,6‐^3^H‐uridine (activity of 1 mCi/mL; Perkin Elmer Inc. Cat# NET‐367) in 7H9 medium was added into each well and incubated the 96‐well plate at 37°C for 72 h. BCG cultures were harvested onto a glass filter paper using an automated cell harvest and radioactivity was measured by using a β counter.

### 2.6 RNA purification and real‐time RT‐PCR

Total RNA was extracted with TRIzol reagent (Sigma‐Aldrich, St. Louis, MO). RNA was converted into cDNA by using a Transcriptor First Strand cDNA Synthesis Kit (Roche). Quantitative real‐time PCR (qRT‐PCR) was performed with SYBR Green (Roche) using the 7500 Real‐Time PCR System (Applied Biosystems). The sequences of primers used in this study were: mouse TNF‐α cDNA: sense: agccgatgggttgtaccttgtcta; antisense: tgagatagcaaatcggctgacggt, mouse iNOS cDNA: sense: gttctcagcccaacaatacaaga; antisense: gtggacgggtcgatgtcac, mouse p40 cDNA: sense: acctgtgacacgcctgaagaagat; antisense: tcttgtggagcagcagatgtgagt, mouse p35 cDNA: sense: acctgctgaagaccacagatgaca; antisense: tagccaggcaactctcgttcttgt, mouse p19 cDNA: sense: tgcaccagcgggacatatgaatct; antisense: tgttgtccttgagtccttgtgggt, and mouse GAPDH cDNA: sense: aactttggcattgtggaagg; antisense: acacattgggggtaggaaca.

### 2.7 Immunoprecipitation/western blots

Cell was lysed by RIPA buffer containing protease inhibitors cocktail (Sigma‐Aldrich, St. Louis, MO) and PMSF (Sigma‐Aldrich, St. Louis, MO). Three hundred micrograms of lysates was precleared by 1 μg of rabbit IgG (Sigma‐Aldrich, St. Louis, MO) together with 40 μL of Protein G PLUS‐Agarose (Santa Cruz), and incubating for 30 min at 4°C. One microgram of anti‐TTP (Sigma‐Aldrich, St. Louis, MO) was added into 1 mL of precleared lysates and incubated for 1 h at 4°C. After 1 h incubation, 40 μL of Protein G PLUS‐Agarose was added and rotated overnight on the rocker platform at 4°C. Immunoprecipitation was collected by centrifugation at 1000 × *g* for 5 min at 4°C and washed 5 times with 1 mL RIPA buffer. Samples were boiled in 40 μL of 1× electrophoresis buffer for 5 min. For western blotting, generally 40–100 μg of whole‐cell lysates were used in SDS–PAGE. SDS–PAGE gels were transferred to PVDF membranes and blocked in 5% nonfat milk in Tris buffer, pH 8.0. PVDF membranes were incubated in the primary antibody with 5% milk/Tris buffer and left overnight at 4°C. After three times washing, PVDF membranes were transferred and incubated with secondary antibody diluted in Tris buffer for another 2 h at room temperature. After three times washing, immune complexes were detected using the SuperSignal West Pico chemiluminescent substrate (Thermo, Rockford, IL).

### 2.8 Statistical analysis

Data were analyzed with Prism software 5.0 (GraphPad). For standard datasets, an unpaired two‐tailed Student's *t*‐test was used. For multiple groups, one‐way ANOVA was used. *: *p* < 0.05; **: *p* < 0.01; ***: *p* < 0.001 indicate significant differences between the compared groups.

## 3 RESULTS

### 3.1 TTP deficiency leads to inhibition of mycobacterial growth and increased expression of proinflammatory cytokines

**FIGURE 1:**
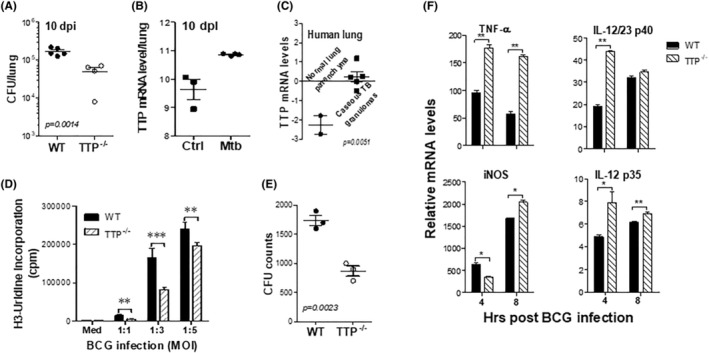
Macrophages deficient in TTP show reduced mycobacterial growth and increased inflammatory cytokines expression. (A) TTP^−/−^ mice and WT littermates aged 5–6 weeks were infected with Mtb via aerosol route (~100 CFU of Mtb Erdman strain per mouse). Ten days post‐infection (dpi), lungs were taken and homogenized in 1 mL of PBS. Serial dilutions of lung homogenates were inoculated onto Middlebrook agar plates and CFUs determined 3 weeks later. Each dot represents one mouse. (B) WT B6 mice aged 6–8 weeks were infected aerosolly with 100 CFU of Mtb as described above. Lung tissues were harvested at 10 dpi and RNA extracted to detect TTP mRNA by real‐ time PCR. (C) Human TB lung tissues were surgically excised from TB patients who had extensive lung cavitation and tissue degeneration with little response to antibiotics. Total RNA was isolated by laser capture microdissection, followed by use of PicoPure™ RNA Isolation Kits. Genome‐wide analysis was performed with GeneChip® Human X3P Array41. (D) BMDMs differentiated from WT and TTP^−/−^ mice were infected with live BCG (MOI = 1, 3, 5). After 48 h, intracellular BCG were measured by radiolabeling with tritiated uridine. Data shown are mean ± SD from four experiments. (E) WT and TTP^−/−^ BMDMs were infected with BCG (MOI = 3). After 48 h, serial dilutions of cell lysates were inoculated onto Middlebrook agar plates and CFUs determined 3 weeks later. Each dot represents BMDMs from one mouse. (F) WT or TTP^−/−^ BMDMs were infected with BCG (MOI = 3). Total RNAs were extracted at 4 and 8 h post‐infection and used to detect mRNAs of the cytokines. Data shown are means plus SD of three to five experiments. Student's *t*‐test were used to compare the difference between WT and KO groups (*p < 0.05; **p < 0.01; ***p < 0.001).

TTP suppresses several early‐response genes in macrophages important for TB clearance, such as iNOS, TNF‐α, and IL‐12. During Mtb infection, macrophages are the front line to encounter the bacteria and initiate immune responses against Mtb infection. Mtb‐specific adaptive immune responses are developed about 3 weeks after aerosol challenge. To define the role of TTP in Mtb growth in vivo, we infected conventional TTP deficient mice and WT littermates with low‐dose Mtb (~100 CFU/per mouse) via the aerosol route, and then measured bacterial burdens at 10 days after infection. In this early phase, innate immune cells, such as macrophages, are major host defense against Mtb infection. As shown in Figure 1A, the numbers of bacteria in lungs were significantly reduced in TTP^−/−^ mice compared with WT littermates. Ten days after infection, very few bacteria were detected in spleens. Next, we analyzed TTP expression in lung homogenates of the Mtb‐infected WT mice. TTP mRNA was increased at 10 dpi (Figure 1B). In humans, TB progression and transmission involves the development of caseous granulomas that cavitate and release infectious Mtb bacilli.^39^, ^40^, ^41^, ^42^ Analyzing a gene dataset in Russell's study,^43^ we found TTP mRNA levels were significantly higher in caseous TB granulomas than normal lung parenchyma (Figure 1C). These data suggest that higher TTP at the early phase of infection may be associated with Mtb infection/activation. To determine whether macrophages are responsible for TTP‐mediated Mtb growth in vivo, we calculated mycobacterial growth in macrophages by measuring intracellular BCG growth by ^3^H‐uridine incorporation which had been verified to correlate with CFU plating.^44^, ^45^ BCG growth was reduced in TTP^−/−^ macrophages compared with WT macrophages infected with different MOIs from 1 to 3 (Figure 1D). The CFU counts were also much lower in TTP^−/−^ macrophages than WT cells (Figure 1E.), suggesting that TTP in macrophages is detrimental and promotes intracellular mycobacterial growth. Several cytokines are important for host defense against Mtb growth, including TNF, iNOS, and IL‐12. Therefore, we measured the expression of these cytokines in TTP^−/−^ macrophages 4 and 8 h after BCG infection. The expression of these cytokines was increased in BCG‐infected TTP^−/−^ macrophages compared with infected WT macrophages (Figure 1F). Taken together, these data indicate that TTP in macrophages is required for optimal mycobacterial growth, mediated probably through suppression of the protective proinflammatory cytokine expression.

### 3.2 Live mycobacteria induce TTP expression in macrophages through the mTOR pathway

**FIGURE 2:**
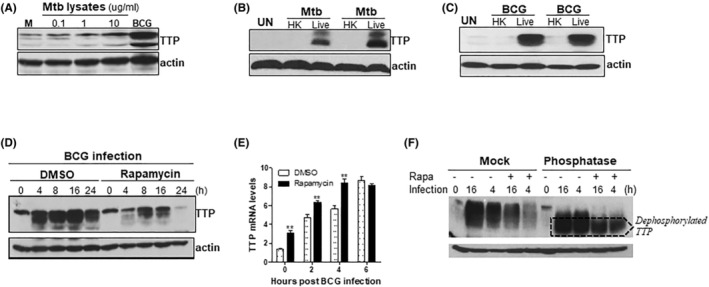
Live mycobacteria induce TTP in macrophages through mTOR pathway. WT BMDMs were treated with 0.1, 1 and 10 μg/mL of Mtb lysates (H37Rv) or infected with live BCG at MOI of 3 (A), treated with heat‐killed (HK) Mtb or live Mtb at MOI of 1 (B), or treated with HK or live BCG at MOI of 3 (C), for 16 h. Whole cell lysates were extracted and TTP protein levels detected by WB. Images shown in (B, C) were from two independent experiments. (D) WT BMDMs were pretreated with DMSO or 1 μM of rapamycin for 2 h and then infected with BCG at MOI of 3. TTP protein levels were detected by WB at 4, 8, 16, and 24 h post‐infection. (E) WT BMDMs were pretreated with DMSO or 1 μM of rapamycin for 2 h and then infected with BCG at MOI of 3. Total RNAs were extracted and TTP mRNAs detected with qRT‐PCR at 2, 4, and 6 h post‐infection. Data shown are means plus SD of three experiments. Student's *t*‐test were used to compare the difference between DMSO and Rapamycin groups at each time point (**: *p* < 0.01). (F) WT BMDMs were pretreated with DMSO or 1 μM of rapamycin for 2 h and then infected with BCG (MOI = 3) for 4 or 16 h, respectively. Whole cell lysates were digested with 70 U phosphatase at 30°C for 2 h. Phosphorylated and dephosphorylated TTP protein levels are detected by WB. Actin served as a loading control. All images shown represent one of three experiments with similar results.

Pathogenic Mtb induces many pathways shared with non‐pathogenic Mtb. However, they also trigger different types of intracellular signaling.^46^ To test whether pathogenic or non‐pathogenic Mtb induced different TTP expression, we first treated macrophages with Mtb lysates and live BCG. Various amounts of Mtb lysates did not induce TTP protein expression while BCG infection did (Figure 2A). We next treated macrophages with both live and heat‐killed (HK) Mtb (Figure 2B) as well as live and HK BCG (Figure 2C), and found that TTP was induced in macrophages only by live mycobacteria, suggesting a unique pathway triggered by live mycobacteria for TTP induction. Viable Mtb activates different metabolisms in host macrophages. The stages of metabolism detected by PET scan are currently used in clinics and clinical trials to monitor TB activity. Mammalian target of rapamycin (mTOR) is one of the main regulators of metabolism and activated by Mtb infection.^32^, ^47^, ^48^, ^49^ To test if the mTOR pathway was involved in TTP induction, we treated BCG‐infected macrophages with rapamycin (mTOR inhibitor) and then checked TTP expression. BCG‐induced TTP protein expression was almost completely inhibited by rapamycin (Figure 2D). In contrast to protein expression, the expression of TTP mRNA was even increased at early time points after rapamycin treatment (Figure 2E), indicating that mycobacteria through the mTOR pathway induce TTP expression at the translational level. During early inflammatory responses, phosphorylation plays a major role in regulating TTP function through affecting both activity and stability of TTP.^50^ The phosphorylation of TTP prevents the proteasome‐dependent degradation of TTP and is accompanied by an increase of the molecular weight of TTP. Using lambda protein phosphates, we found that dephosphorylated TTP was still presented after rapamycin treatment (Figure 2F), suggesting that rapamycin‐mediated TTP inhibition does not affect its phosphorylation. Overall, these results indicate that live mycobacteria induce TTP expression in macrophages through the mTOR signaling pathway.

### 3.3 mTOR‐C1 mediates BCG‐induced TTP induction in macrophages

**FIGURE 3:**
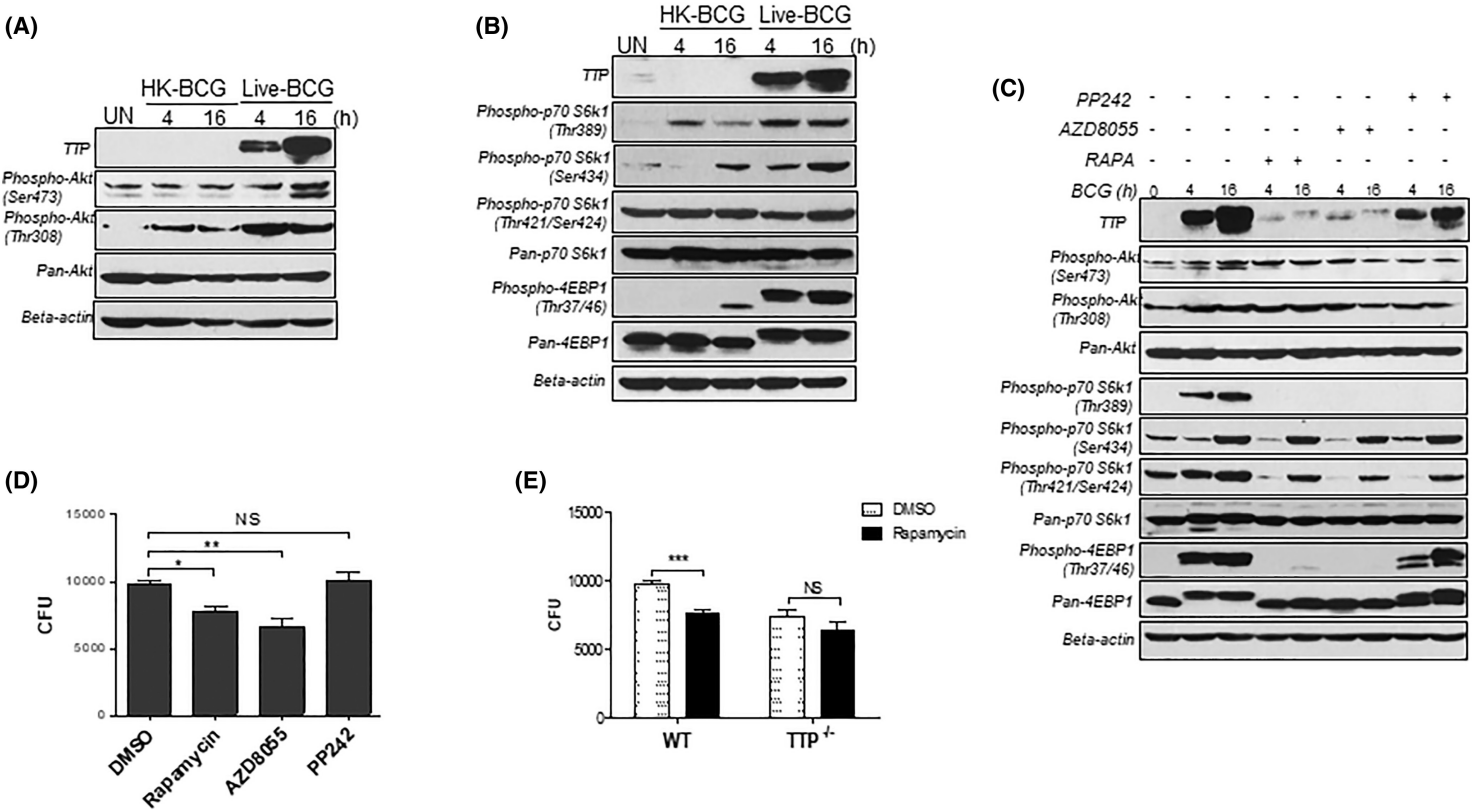
Activated mTOR‐C1 signaling induced by mycobacteria mediates TTP induction and function. (B) WT BMDMs were infected with HK or live BCG at MOI of 3. Phospho‐Akt (Ser473), phospho‐AKT (Thr308), total AKT protein (A), as well as phospho‐p70 S6K1 (Thr389), phospho‐p70 S6K1 (Ser434), phospho‐p70 S6K1 (Thr421/Ser424), total p70 S6K1, phospho‐4EBP1 (Thr37/46), and total 4EBP1 protein levels (B) were detected by WB at 4 and 16 h post‐infection. (C) WT BMDMs were pretreated for 2 h with 1 μM of rapamycin, 1 μM of AZD8055 or 2 μM of pp242, and then infected with BCG at MOI of 3. Phospho‐Akt (Ser473), phospho‐AKT (Thr308), total AKT, phospho‐p70 S6K1 (Thr389), phospho‐p70 S6K1 (Ser434), phospho‐p70 S6K1 (Thr421/Ser424), total p70 S6K1, phospho‐4EBP1 (Thr37/46), and total 4EBP1 protein levels in BMDM were detected by WB at 4 and 16 h post‐infection. (D) WT BMDMs were pretreated for 2 h with 1 μM of rapamycin, 1 μM of AZD8055 or 2 μM of pp242, and then infected with BCG at MOI of 3 for 1 h and washed three times. Cells are lysed after 48 h of infection and CFUs were enumerated on Middlebrook agar plate after 3 weeks of culture. (E) WT and TTP^−/−^ BMDMs were pretreated for 2 h with 1 μM of rapamycin or DMSO, and then infected with BCG (MOI = 3). After 48 h, serial dilutions of cell lysates were inoculated onto Middlebrook agar plates and CFUs counted 3 weeks later. Data shown in D & E are means plus SD of three experiments. Student's *t*‐test were used to compare the difference between indicated groups (*: *p* < 0.05; **: *p* < 0.01; ***: *p* < 0.001). NS: no statistical differences.

The mTOR signaling pathway is a central regulator of cell metabolism, growth, proliferation, and survival.^22^ To further determine the role of mTOR signal pathway in TTP expression, we checked three canonical downstream targets of mTOR, Akt, P70‐S6K1, and 4EBP1. The phosphorylation site of Akt at ser473 showed little phosphorylation in HK‐BCG‐treated macrophages whereas its levels were increased at 16 h after BCG infection (Figure 3A). Although the levels of phosphorylated Akt at thr308 were increased in HK‐BCG‐treated macrophages, BCG infection induced much higher levels of phosphorylated Akt at thr308 than HK‐BCG (Figure 3A). The phosphorylation of Akt ser473 is closely related to activation of the mTOR‐C2 signal pathway, while the phosphorylation site thr308 can be selectively activated by PDK1.^51^ Next, we checked the levels of phosphorylated p70‐S6K1 and 4EBP1. As shown in Figure 3B, the phosphorylated p70‐S6K1 at sites thr389, ser434, and thr421/ser424 were induced by both HK‐BCG and live‐BCG in macrophages, with the sites at thr421/ser424 constitutively phosphorylated in macrophages. Interestingly, the phosphorylated 4EBP1 at site thr37/46 was only induced strongly by BCG infection but not HK‐BCG stimulation (Figure 3B). Taken together, these data indicate that activated mTOR‐C1 signaling molecules AKT and 4EBP1 are correlated with TTP induction in BCG‐infected macrophages.

To further determine whether mTOR‐C1 or mTOR‐C2 mediated BCG‐induced TTP expression, we used three different mTOR‐specific inhibitors, rapamycin (mTOR‐C1 inhibitor), AZD8055, and PP242 (pan mTOR‐C1/C2 inhibitors). As shown in Figure 3C, rapamycin and AZD8055 significantly inhibited TTP induction, whereas pp242 has little effect on TTP expression. Both AZD8055 and pp242 inhibited phosphorylated Akt (ser473 and thr308 sites) expression, while mTOR‐C1 inhibitor rapamycin failed to do so. Phosphorylation of p70‐S6K1 at thr389 is reported to be stimulated by growth factors such as insulin, EGF, and FGF, as well as by serum and some G‐protein‐coupled receptor ligands, and can be blocked by wortmannin, PI‐3 K inhibitor, and rapamycin.^52^ We found that all three inhibitors completely blocked the phosphorylation of p70‐S6K1 at sites thr389 induced by BCG infection and had little effects on phosphorylation of p70‐S6K1 at sites ser434 and thr421/ser424. Rapamycin and AZD8055 completely blocked the phosphorylation of 4EBP1 at sites thr37/46, while pp242 did not block the phosphorylation (Figure 3C). These data indicate that mTOR‐C1 likely mediates BCG‐induced TTP expression in macrophages. To further confirm the function of mTOR pathway in control of mycobacterial growth, we treated BCG‐infected macrophages with these three mTOR inhibitors and then compared the CFUs with the cells treated with DMSO. As shown in Figure 3D, rapamycin and AZD8055 significantly inhibited BCG growth, while pp242 did not, a pattern correlated with their effects on TTP expression shown in Figure 3C. In addition, the rapamycin‐mediated inhibition of BCG growth was abolished in macrophages deficient in TTP (Figure 3E). Overall, these data indicate that mycobacteria induce TTP expression through the mTOR‐C1 signaling pathway, leading to an increase in bacterial growth in macrophages.

### 3.4 mTOR pathway activated by mycobacteria prevents TTP protein degradation

**FIGURE 4:**
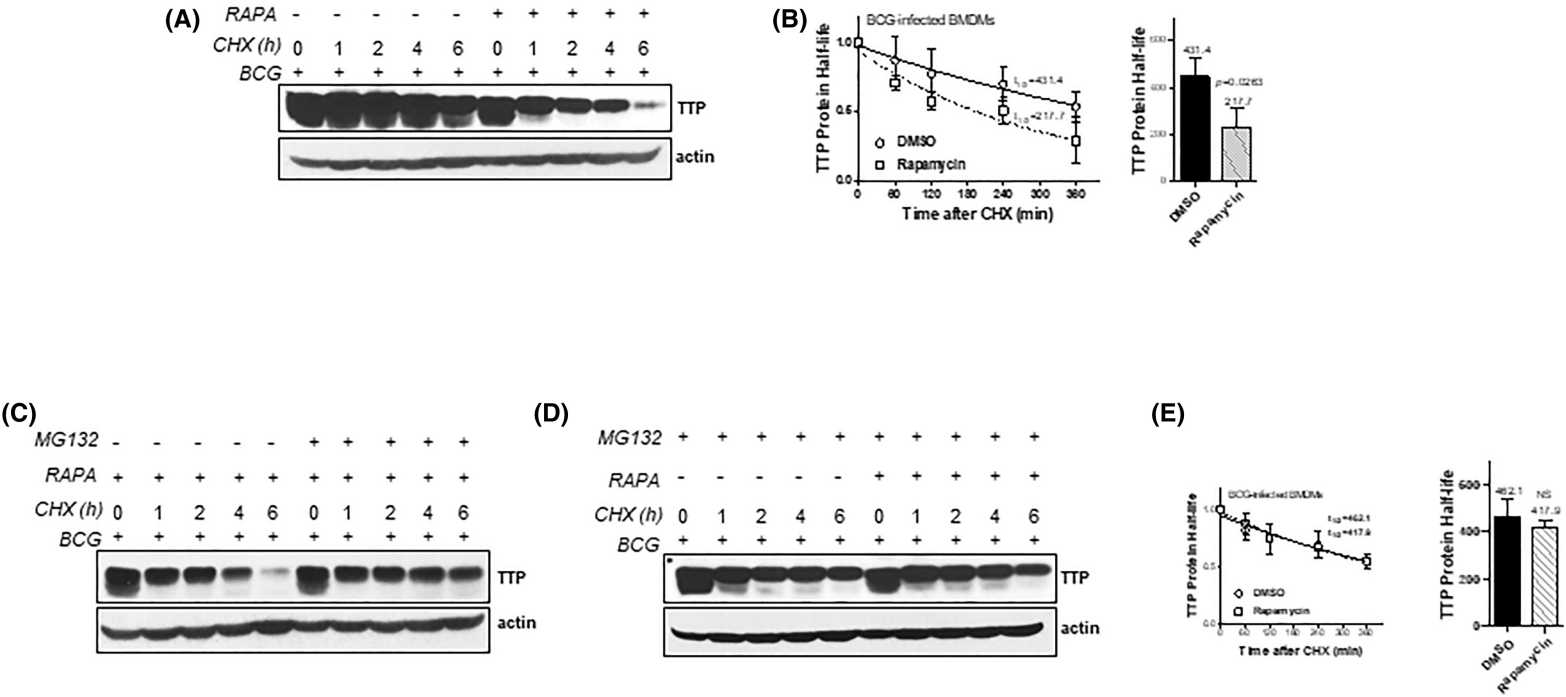
mTOR inhibitor rapamycin destabilizes TTP protein. WT BMDMs were pretreated for 2 h with DMSO or 1 μM of rapamycin and then infected with BCG at MOI of 3 for 16 h. Infected BMDMs were washed once and treated with 20 μg/mL of cycloheximide (CHX) to block de novo protein synthesis. Whole cell lysates were extracted at 1, 2, 4, and 6 h post‐CHX treatment and TTP protein detected with WB (A). The half‐life of TTP protein was calculated with grayscale ratio from three independent experiments using one‐phase decay analysis (B). (C) WT BMDMs were pretreated for 2 h with 1 μM of rapamycin and then infected with BCG (MOI = 3) for 16 h. Infected BMDM was washed once and treated with 20 μg/mL of CHX plus or minus 5 μM of MG132. Whole cell lysates were extracted at 1, 2, 4, and 6 h post‐CHX treatment and TTP protein detected with WB. WT BMDMs were pretreated for 2 h with DMSO or 1 μM of rapamycin and then infected with BCG at MOI of 3 for 16 h. Infected BMDM was washed once and treated with both 20 μg/mL of CHX and 5 of μM of MG132 concurrently. Whole cell lysates were extracted at 1, 2, 4, and 6 h post‐CHX and ‐MG132 treatments and TTP protein detected with WB (D). The half‐life of TTP protein was calculated with grayscale ratio from three independent experiments using one‐phase decay analysis (E).

Blocking the mTOR signaling pathway almost completely abolished TTP protein expression, whereas had no effect on expression of TTP mRNA (Figure 2E), indicating that mTOR regulates TTP at the translational level. Thus, we blocked new protein synthesis with cycloheximide in BCG‐infected macrophages treated with or without rapamycin, and then checked TTP protein. The levels of TTP protein were quickly reduced in rapamycin‐treated macrophages compared with untreated macrophages (Figure 4A). More importantly, the half‐life of TTP protein was significantly reduced from 431.4 ± 75.1 min in untreated macrophages to 217.7 ± 79.9 min in rapamycin‐treated macrophages (Figure 4B). To further confirm mTOR‐mediated TTP protein degradation, we treated the infected macrophages with proteasome inhibitor MG132 prior to adding rapamycin. Rapamycin‐mediated TTP protein degradation was completely reversed by MG132 (Figure 4C). Moreover, the levels of TTP protein in rapamycin‐treated macrophages were recovered to the levels of TTP in DMSO‐treated macrophages (Figure 4D), so does the shortened half‐life of TTP protein (Figure 4E). These findings suggest that the BCG‐activated mTOR pathway enhances TTP protein levels in macrophages through stabilizing TTP protein.

### 3.5 mTOR mediates TTP protein stability through ubiquitin/proteinase machinery

**FIGURE 5:**
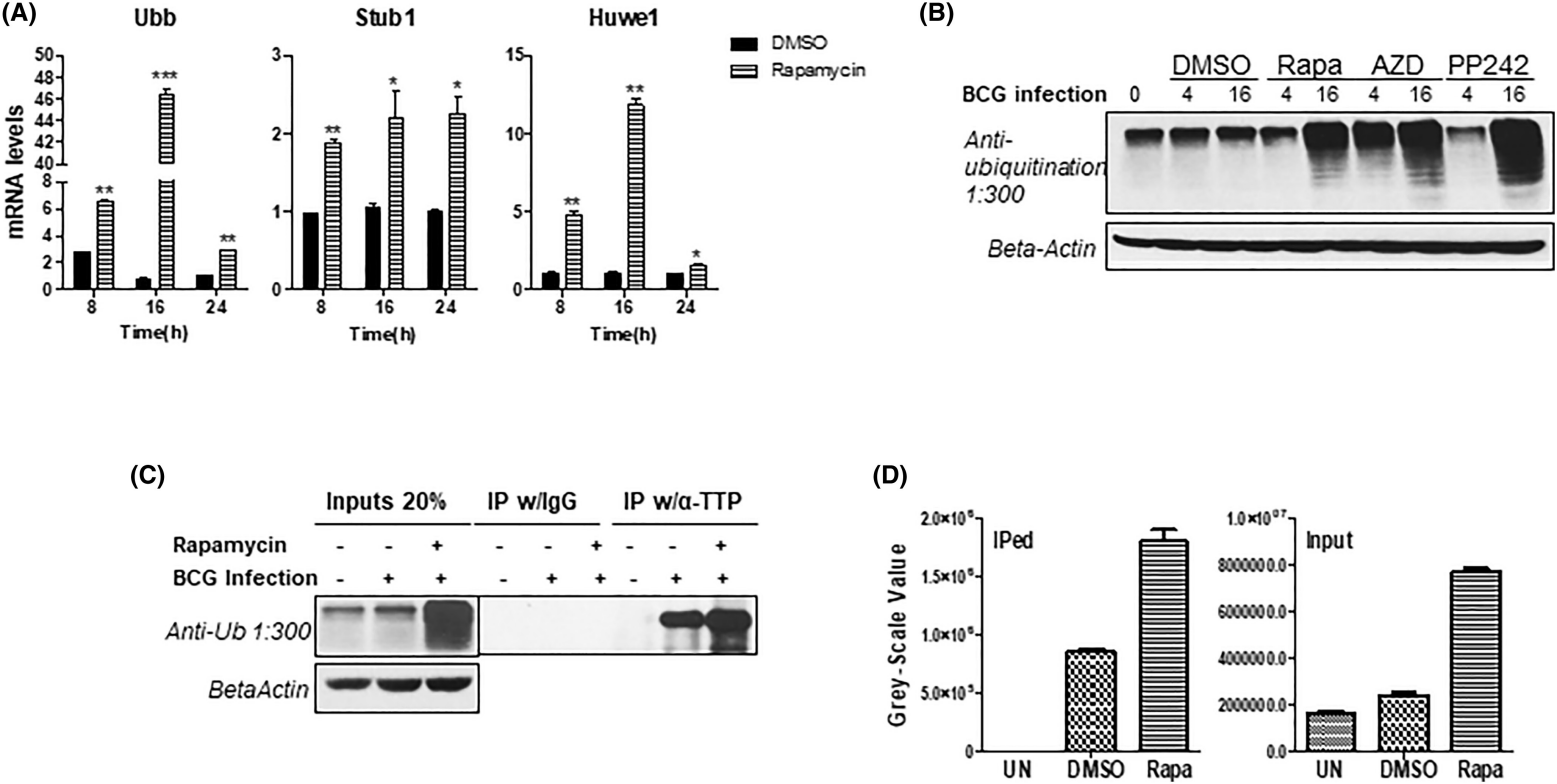
mTOR‐mediated TTP degradation acts through the ubiquitin‐proteasome pathway. (A, B) WT BMDMs were pretreated for 2 h with DMSO or 1 μM of rapamycin and then infected with BCG at MOI of 3. Ubb and Stub1 mRNAs were detected by qRT‐PCR at 8, 16, and 24 h post‐infection. qRT‐PCR data shown are means plus SD from three independent experiments and normalized relative to GAPDH mRNA expression levels in each sample and further normalized to the sample from the cells treated with DMSO (*: *p* < 0.05; **: *p* < 0.01; ***: *p* < 0.001). (C) WT BMDMs were pretreated for 2 h with DMSO, 1 μM of rapamycin, 1 μM of AZD8055 and then infected with BCG at MOI of 3. Whole‐cell ubiquitinated protein levels at 4 and 16 h post‐infections were quantified with anti‐ubiquitination antibody by WB. Actin served as a loading control. The images shown represent one of three experiments. WT BMDMs were pretreated with DMSO or 1 μM of rapamycin for 2 h and then infected with BCG (MOI = 3). 5 μM of MG132 was added to infected BMDM to prevent TTP degradation. After 16 h, whole cell lysates were extracted and immunoprecipitated with specific antibody against TTP and isotype control IgG. The IPed ubiquitinated TTP was detected with anti‐Ub antibody (1:300 dilution) by WB (D). Relative quantitation of TTP ubiquitination was calculated by grayscale ratio as means plus SD of the gray scales from three experiments.

Ubiquitination is an important mechanism for protein regulation in eukaryotic cells.^53^ Proteinase digestion is the final step of protein ubiquitination process, which degrades ubiquitin tagged protein in 26S proteasome.^54^ To test whether ubiquitin‐dependent mechanism contributed to rapamycin‐mediated TTP degradation, we checked the effects of rapamycin on three ubiquitination components Ubb, Stub1, and Huwe1, encoding ubiquitin B and an E3‐ubiquitin protein ligase, respectively. Inhibition of mTOR by rapamycin significantly increased the transcripts of Ubb, Stub1, and Huwe1 in BCG‐infected macrophages (Figure 5A). The mRNA levels of Ubb and Huwe1 reached the peak at 16 h after infection and were approximately 40 and 12 times higher than the untreated cells, respectively (Figure 5A). Meanwhile, Stub1 mRNA levels were kept at constantly high levels in response to rapamycin treatment (Figure 5A). Next, we checked the effects of different mTOR inhibitors on protein ubiquitination in response to BCG infection. Inhibition of mTOR by C1‐specific inhibitor rapamycin or C1/C2 pan‐inhibitor AZD8055 was associated with an increase in ubiquitinated proteins in macrophages after BCG infection (Figure 5B). To further confirm the roles of ubiquitination in TTP degradation, we checked the ubiquitinated form of TTP with co‐immunoprecipitation assay with an antibody specific recognizing TTP. Inhibition of mTOR with rapamycin led to a significant increase in ubiquitinated TTP (Figure 5C,D). These results indicate that inhibition of the mTOR signaling pathway promotes TTP degradation through a ubiquitin/proteinase‐dependent mechanism.

### 3.6 Blocking TTP binding increases expression of the protective cytokines and suppresses intracellular BCG growth

**FIGURE 6:**
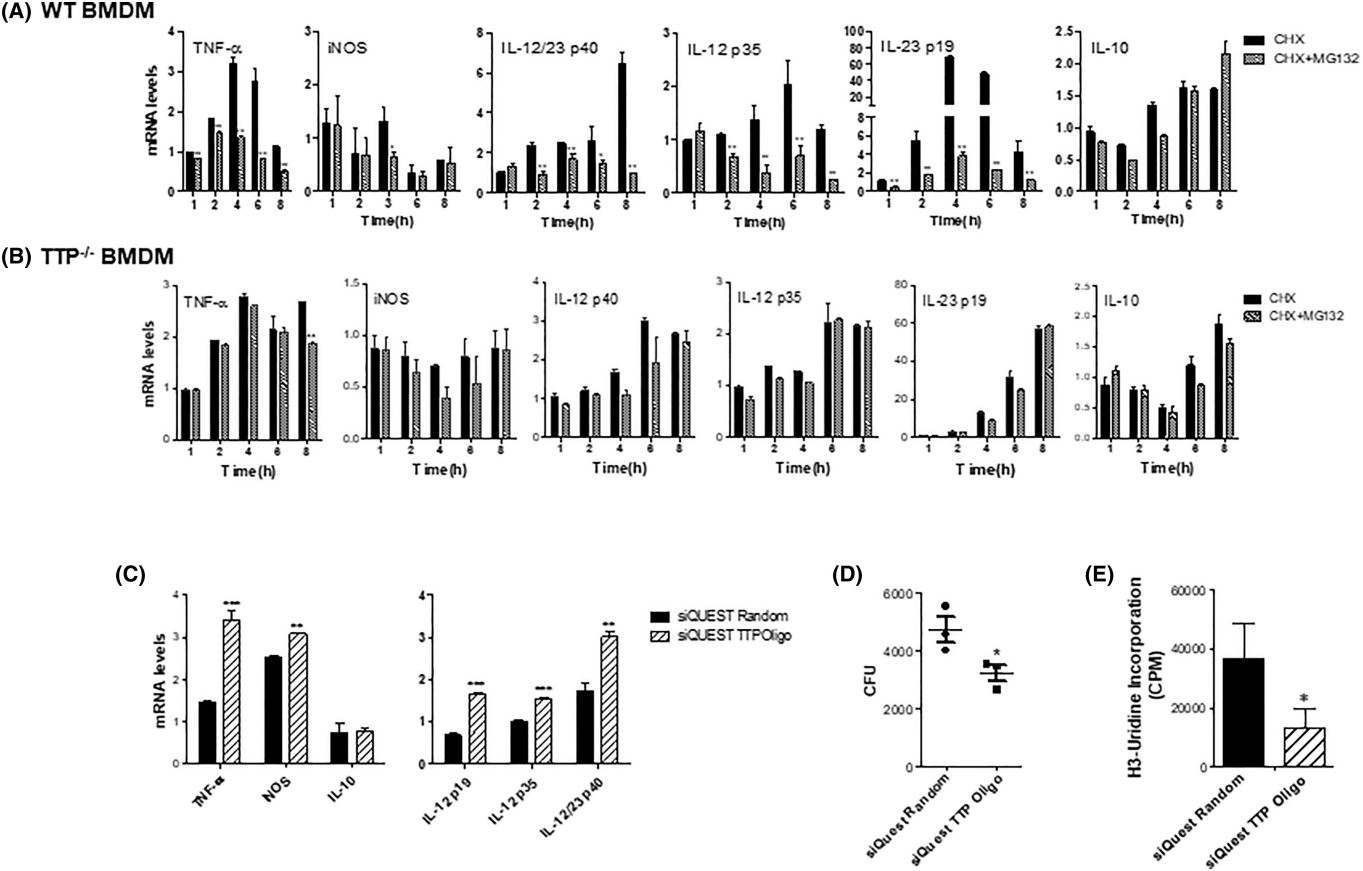
Blocking TTP binding suppresses BCG growth and increase expression of the cytokines critical for Mtb clearance in macrophages. (A, B) WT and TTP^−/−^ BMDMs were infected with BCG at MOI of 3 and treated with 20 μg/mL of CHX with or without 5 μM of MG132. The mRNA profiles of cytokines at 1, 2, 4, 6, and 8 h post‐BCG infection were detected by qRT‐PCR. (C). RAW264.7 cells were transfected with ARE analog TTP Oligo or random oligo sequence 2 h prior to BCG infection (MOI = 3). The mRNA profiles of the indicted cytokines were detected at 2 h post‐BCG infection by qRT‐PCR. RAW264.7 cells were transfected with ARE analog TTP Oligo (UUAUUUAUUUAUU) or random oligo sequence 2 h prior to BCG infection (MOI = 3). After 2 h, cells were washed three times and lysed. CFUs were enumerated on Middlebrook agar plate after 3 weeks of culture (D). The lysates form the above cells were used to measure intracellular BCG by radiolabeling with tritiated uridine (E). qRT‐PCR data shown are means plus SD from three independent experiments and normalized relative to GAPDH mRNA expression levels in each sample and further compared the cells treated with CHX to the cells with CHX + MG132 (A, B) or compared the cells transfected with and without TTP oligo (C). *p < 0.05; **p < 0.01; ***p < 0.001.

It has been reported by us and others that TTP targets inflammatory cytokine expression, including TNF‐α, iNOS, IL‐12/IL‐23 p40, IL‐12 p35, IL‐23 p19, and IL‐10. Many of these cytokines are important for host defense against Mtb infection. To test whether TTP was responsible for cytokine suppression in BCG‐infected macrophages, we blocked de novo protein synthesis with CHX and prevented proteinase activity with MG132. As shown in Figure 6A, blocking protein degradation led to an inhibition in expression of these protective cytokines. However, the inhibitory effects were almost completely abolished in macrophage deficient in TTP (Figure 6B), further confirming the roles for TTP protein in immune suppression by mycobacteria in macrophages.

It is known that TTP destabilizes mRNAs of the target cytokines by binding to the consensus AREs regions. To test whether blocking binding of TTP to its target mRNAs enhanced expression of protective cytokines, such as TNF‐α, iNOS, and IL‐12 family cytokines, we synthesized a consensus sequence of the ARE (UUAUUUAUUUAUU) which could competitively bind to endogenous TTP and therefore prevent it from destabilizing target mRNAs during infection. Transfection of the core consensus ARE sequence into macrophages significantly increased the expression of mRNAs encoding TNF‐α and iNOS, as well as the mRNAs encoding IL‐12/IL‐23 p40, IL‐12 p35, and IL‐12 p19 during BCG infection, while had little effect on IL‐10 mRNA (Figure 6C). Next, we tested the effects of blocking TTP binding on intracellular mycobacterial growth. Blocking TTP binding with the consensus ARE sequences strongly suppressed BCG growth in macrophages compared with the cells transfected with the random sequences (Figure 6D,E). These results indicate that ARE sequence analog can prevent mRNA degradation of the protective proinflammatory cytokines mediated by TTP, thus increasing host protective immune responses against mycobacteria in macrophages. TTP could be a promising target for host‐directed therapy against Mtb infection.

## 4 DISCUSSION

Upon inhalation, Mtb is captured by alveolar macrophages and triggers early inflammatory immune responses. The recognition of Mtb structural components through TLRs activates intracellular signaling cascades of proinflammatory biosynthesis in macrophages.^55^ However, the intensity of inflammatory responses is subject to the control of mRNA stability, which mediated mainly by a set of RBPs, including one of the best characterized RBP TTP.^56^ TTP plays a critical role in resolution of inflammation through destabilizing the proinflammatory cytokines important for Mtb clearance. However, the role of TTP in TB is poorly understood. Deficiency in TTP resulted in low bacterial burden in lungs of the Mtb‐infected mice (Figure 1A), which is in line with the increased TTP in human patients with active TB (Figure 1C). TTP‐deficient macrophages displayed similar inhibitory effects on mycobacterial growth as the TTP knockout mice 10 dpi, indicating a role for TTP in regulating early immune responses to Mtb infection. At 10 dpi, the adaptive immune responses, such as Mtb‐specific Th1 cells, are not fully developed; and host immune responses are mainly mediated by innate immune cells. Due to the lower number of bacteria in the lung, the number of bacteria disseminated to distant organs, such as spleen, are rare and undetectable in this study. The increased inflammatory cytokines in TTP‐deficient macrophages (Figure 1F) could enhance bactericidal effects, resulting in lower CFUs in lungs of the infected mice and in macrophages after infection. TTP protein was induced by live but not dead mycobacteria in macrophages (Figure 2A–C), suggesting that mycobacteria facilitate their growth in host macrophages through suppressing protective immune responses via inducing TTP. Besides Mtb, other infectious pathogens can also induce TTP. Human herpesvirus type 1 (HSV‐1) can induce TTP expression in host cells.^57^ A viral protein of HSV‐1 virion host shutoff (VHS) can physically bind with TTP CCCH motif and mRNA cap structure to mediate the degradation of AU‐rich mRNAs and selectively degrade the host macromolecules which are harmful to viral replication.^58^ Thus, the TTP induction by various pathogens may be a universe mechanism for pathogens to evade host protective immune responses.

mTOR, a serine threonine kinase, integrates numerous stimuli and directs cellular decisions. Gutierrez et. al first reports that inhibition of mTOR by rapamycin enhances Mtb clearance in macrophages through induction of autophagy.^30^ Autophagy can be induced by pattern recognition receptors and participates in eliminating intracellular pathogens through directly digesting intracellular microorganisms, promoting surface antigen presentation, and facilitating the synthesis of secretory proteins.^59^, ^60^, ^61^ Later, it was found that inhibition of mTOR can enhance host defense against a wide range of intercellular pathogens from bacteria, virus to protozoa.^27^, ^28^, ^29^, ^33^, ^34^ The role of mTOR in TB, however, is complicated. Jagannath et al. reports that rapamycin enhances Mtb defense by promoting antigen presentation, Th1 type immune responses and DC‐mediated vaccine efficacy.^31^ Administration of rapamycin also displays significant effects against intracellular Mtb in macrophages and a reduction in Mtb burdens in combination with anti‐TB treatment in vivo.^62^, ^63^ The zebrafish deficient in mTOR are more likely to clear infection early. Similar results are also observed with rapamycin analogues, nitazoxanide (an FDA‐approved antiparasitic) and its active form tizoxanide.^64^ Most of these effects are believed to be associated with autophagy induction by mTOR inhibition. However, recent findings challenge this theory. Knockout key molecules in autophagy formation did not affect bacterial growth in mice infected with Mtb,^65^ suggesting additional mechanisms besides autophagy contributing to the anti‐TB activity of mTOR inhibition. In this study, we found that inhibition of mTOR with rapamycin almost completely abolished TTP induction by Mtb and BCG (Figure 2D), along with an inhibition in bacterial growth in macrophages (Figure 3D) to similar extent as the macrophages deficient of TTP (Figure 3E). Consistent with our findings, BCG infection activated the Akt/mTOR pathway and phosphorylated two major downstream effectors 4EBP and S6K1 (Figure 3A,B). Although phosphorylated S6K1 (Thr389) was completely blocked by all three mTOR inhibitors (rapamycin, AZD8055, and pp242), only phosphorylated 4EBP1 (thr37/46) correlated with TTP inhibition (Figure 3C). Since rapamycin is specifically targeting mTOR‐C1, it seems that the mTOR‐C1/4EBP1 signaling pathway medicates TTP induction by mycobacteria. Collectively, these results reveal a new mechanism for the anti‐TB function of mTOR inhibition via TTP suppression.

Rapamycin inhibited TTP protein but mRNA expression, which suggests translational regulation. Indeed, rapamycin promotes TTP protein degradation by shorting TTP protein half‐life about twofold (Figure 4A,B). Blocking proteasome activity with MG132 almost completely recovered the shortened TTP half‐life induced by rapamycin to the levels in untreated cells (Figure 4C–E). The increased TTP mRNA after rapamycin treatment could be negative feedback as a result of the reduced TTP protein. It is known that the mRNA of TTP is also targeted for degradation by itself.^66^ TTP protein degradation by rapamycin is mediated through ubiquitin machinery, probably through Ubb and Stub1 subunits as well as Huwe1 which shown to control TTP proteasomal degradation^67^ (Figure 5). It has been reported that rapamycin treatment increases the expression of proinflammatory cytokines, including TNF‐α, IL‐6, IL‐12, IL‐23, in Mtb‐infected monocytes/macrophages.^23^, ^24^ How these cytokines are induced by rapamycin is still not completely understood. Since blocking proteasome activity with MG132 stabilized and enhanced TTP protein in BCG‐infected macrophages, we boosted TTP protein level in BCG‐infected macrophages by treating the cells with CHX plus MG132. These treatments resulted in inhibition of proinflammatory cytokine TNF‐α, iNOS, IL‐12, IL‐23, but not IL‐10 (Figure 6A). Importantly, the inhibition is abolished when TTP is knocked out (Figure 6B), further supporting the concept that rapamycin through TTP regulates the expression of these cytokines. Since IL‐1,^68^ IL‐6,^69^ IFN‐γ^30^, ^70^ and TNF‐α^71^, ^72^, ^73^ promote autophagy formation in macrophages, induction of TTP by mycobacteria through the mTOR pathway can suppress expression of these cytokines and therefore inhibit autophagy formation, leading to an increase in bacterial growth. Moreover, the suppression of TTP by mTOR inhibitor rapamycin also gives us a clue of the paradoxical immunostimulatory effects of the mTOR inhibitors.^74^ Patients treated with rapamycin as an immunosuppressant show an enhancement of antigen presentation, IL‐12 signaling activation, and increased inflammatory and immune responses.^75^ Patients can benefit from the immunostimulatory effects. Several clinical trials have proved systematic use of mTOR inhibitors enhances immune function, reduces infections in the elderly, and decreases the risk of CMV and polyomavirus infection in solid organ transplant recipients.^76^, ^77^ Blocking TTP binding with decoy ARE sequence effectively increases the expression of TNF‐α/iNOS/IL‐12/IL‐23, and suppresses mycobacterial growth in macrophages (Figure 6 C–E). Collectively, the mTOR signaling pathway and RBP TTP could be promising therapeutic targets in infectious disease especially in Mtb infection. Wild‐type C3HeB/FeJ mice are sensitive to Mtb infection, and it is a valuable model system to evaluate host‐derived therapy candidates targeted against Mtb infection. Kamlesh Bhatt et al used the C3HeB/FeJ mice to investigate the role of rapamycin as an adjunctive therapy candidate during the treatment of Mtb infection with moxifloxacin. They reported that administration of rapamycin with or without moxifloxacin reduced infection‐induced lung inflammation, CFU, and the number and size of caseating necrotic granulomas.^78^ Our study reveals a new mechanism for the anti‐TB effects of Rapamycin through inhibiting TTP expression and TTP‐mediated immune escape and Mtb progression.

## AUTHOR CONTRIBUTIONS

Concept and design: J.L. Development of methodology: J.L., J.W. and H.N. Acquisition of data (provided animals, plasmids and reagents, etc.): J.W., H.N., Q.W. and C.E. Analysis and interpretation of data: J.L., J.W., H.N. and C.E. Writing, review, and/or revision of the manuscript: J.L., J.W., H.N., O.R.‐E., R.H., M.F., D.F., E.Y.L. and D.F.H. Study supervision: J.L.

## DISCLOSURES

The authors declare no competing financial interests.

## Data Availability

Figure 1C data were derived from the following resources at https://pubmed.ncbi.nlm.nih.gov/20597103/.^43^ The data that support the findings of this study are available in the methods of this article.
